# Contributing factors to unsafe abortion practices among women of reproductive age at selected district hospitals in the Ashanti region of Ghana

**DOI:** 10.1186/s12905-019-0759-5

**Published:** 2019-05-03

**Authors:** Confidence Alorse Atakro, Stella Boatemaa Addo, Janet Sintim Aboagye, Awube Menlah, Isabella Garti, Kwaku Gyimah Amoa-Gyarteng, Theresa Sarpong, Peter Adatara, Kwasi Junior Kumah, Bernard Bediako Asare, Ami Korkor Mensah, Squiter Hans Lutterodt, George Sedinam Boni

**Affiliations:** 1 Queensland University of Technology, Brisbane, Australia; 2 Scool of Nursing and Midwifery, Christian Service University College, Kumasi, Ghana; 3 Valley View University, Accra, Ghana; 4 Suntreso Government Hospital, Kumasi, Ghana; 5 School of Nursing, University of Health and Allied Sciences, Ho, Ghana; 6 Seventh Day Adventist Hospital, Asamang, Ghana; 7 Volta Regional Hospital, Ho, Ghana

**Keywords:** Unsafe, Abortion, Ghana, Law, Taboo, Stigma, Unplanned, Abortion policy, Ashanti

## Abstract

**Background:**

Despite the existence of an abortion law and a safe abortion policy in Ghana, the Ghana Statistical Service found that 15% of all women in the reproductive age group (15–49 years) have practiced unsafe abortions. The objective of this study was to explore factors that contribute to the high incidence of unsafe abortion practices in the Ashanti Region of Ghana.

**Methods:**

A qualitative descriptive study design was used to assess factors that contribute to unsafe abortion practices. Purposive sampling technique was employed in selecting participants. Data were collected through key informant interviews and focus group discussions. One hundred and eleven participants were involved in the study. Data analysis was carried out through qualitative content analysis.

**Results:**

Seven thematic categories were elicited from data collected. The categories are: a) Lack of knowledge of safe abortion services; b) Socio-economic conditions as a perceived influence for unsafe abortion practices; c) Safe abortion as a perceived religious and cultural taboo in Ghana; d) Stigma of unplanned pregnancy; e) A desire to bear children only after marriage; f) Avoiding parental/guardian disappointment and resentment; g) A desire to pursue education.

**Conclusions:**

Evidence available in this study suggests that several factors are responsible for unsafe abortion practices in Ghana. Lack of knowledge on safe abortion services, poor socio-economic conditions, cultural and religious beliefs, a stigma of unplanned pregnancy, a desire to bear children only after marriage, attempts to avoid parental/guardian disappointment and resentment, and a desire to pursue education were cited by participants as situations that contributed to unsafe abortion practices. Measures such as Aunty Jane, Ms. Rose and Women Help Women programmes can be publicised to reduce maternal morbidity and mortality that occur as a result of unsafe abortions in Ghana. Improvement in family planning education in educational institutions needs to be considered in order to reduce the rate of unwanted pregnancies among young women in school.

**Electronic supplementary material:**

The online version of this article (10.1186/s12905-019-0759-5) contains supplementary material, which is available to authorized users.

## Background

The World Health Organization (WHO) defined unsafe abortion as a procedure for terminating a pregnancy performed by persons lacking the necessary skills or in an environment, not in conformity with minimal medical standards, or both [1]. About 97% of all global abortions between 2010 and 2014 occurred in low income countries [2]. The percentage of unsafe abortions are higher in countries with highly restrictive abortion laws when compared with countries with more liberal laws [2]. However, it must be noted that even in countries where abortion laws are liberal [3], some women still rely on unsafe abortions because of existing additional barriers such as onerous facility requirements, waiting periods, and parental consent laws [4]. Though evidence available shows that self-management of abortions with the use of abortions pills (misoprostol alone or misoprostol in combination with mifepristone) can reduce maternal mortality and morbidity, inadequate information can lead to wrong dosages which will subsequently end in unsafe abortions [1]. All abortions in West Africa were found to be unsafe in 1995, 2003 and 2008 [3]. Africa is currently burdened with many abortion-related complications [5]. Maternal mortality is an un-abating problem in less resourced countries [6]. Deaths from unsafe abortions have been discovered to contribute 14% of all maternal deaths in Africa [1, 7]. Apart from the contributions of unsafe abortions to maternal mortality, unsafe abortion practices also result in other health problems. An estimated five million women from less developed countries are hospitalised every year for unsafe abortion complications such as haemorrhage, infections and perforations [8, 9]. The negative effects of unsafe abortions are disproportionately higher in Africa [10–12]. Unsafe abortion is a key proximate cause of maternal mortality in Ghana [13, 14]. Fifteen percent (15%) of all women in the reproductive age group (15–49 years) were found in a national survey to have sought unsafe abortions [15]. Abortion rates in Ghana differ from region to region. A study in by Mote et al. [16] reported a rate of 21.3% from the Volta Region, while another study reported 22.6% in the Brong Ahafo Region [17]. The complications associated with unsafe abortions have serious public health implications for Ghana as they increase maternal mortality and morbidity and divert limited health resources [15]. The Government of Ghana had taken steps to reduce the effects of unsafe abortions by providing a safe abortion policy but the expected decline in maternal deaths is yet to be realised [6]. Since 1985, the abortion law in Ghana has seen some improvements. Abortion is now permitted in law provided it is carried out by registered medical practitioners in registered facilities and where a pregnancy is the result of rape, incest, its continuation would result in injury to a woman’s physical or mental health, or the foetus has a substantial risk of a serious abnormality [18]. Evidence available shows that task shifting of safe abortion services reduces maternal morbidity and mortality [1]. Though some previous researchers have described abortion law in Ghana as progressively liberal [19, 20] in comparison to other African countries such as Nigeria, Côte d’Ivoire and Mali, it is restrictive when compared to other countries such as Canada, United States of America (USA) and Uruguay [21]. Some researchers in Ghana have identified obstacles that continue to hinder the implementations of the abortion law and policy that is aimed at reducing unsafe abortions in Ghana. Aniteye and Mayhew [19] found that despite the existence of safe abortion policy in Ghana, many health workers and facilities do not see the urgency for its implementation because the concept of abortion as sin seems to be pervasive [19]. Ghana can learn from Uruguay which is the first country in South America to recognize the right to abortion on broad grounds [21]. Abortion reforms in Uruguay have been the focus of several studies and advocacy [21]. More studies and similar advocacies are needed in Ghana to improve the practice of safe abortion services to the public. Although the Ghanaian Government has signed the Maputo protocol which states that abortion is a human right for women in Africa [22], it is yet to fully implement policies such as medical abortion services across health facilities in Ghana. Majority of abortion studies in Ghana were quantitative studies and literature reviews [5, 10, 16, 17, 19, 22]. Earlier studies in Ghana concluded that there was inadequate qualitative research regarding unsafe abortions [6, 19]. Other researchers [23, 24] also concluded in their studies that there was a dearth of relevant information existing on the current practices of unsafe abortion and operations of unsafe abortion services. A review of abortion care in Ghana by Rominski and Lori [6] recommended that there was a need to gather information from women regarding their experiences in securing safe and legal abortions as well as reasons for resorting to unsafe methods. Rominski and Lori [6] indicated that these experiences of women will enable policymakers to pinpoint interventions that will prevent maternal mortality and other life-threatening complications that occur as a result of unsafe abortions. This study used qualitative approaches where key informant interviews and focus group discussions [FGDs] were conducted to assess contributing factors to unsafe abortion practices in the Ashanti Region, which has the second highest percentage (20.8%) of women who have ever attempted unsafe abortions [15].

## Methods

### Design

Qualitative descriptive design was used to assess factors that contributed to unsafe abortion practices among women of the reproductive age group in the Ashanti Region of Ghana. Key informant interviews and focus group discussions were utilised in this study. The qualitative descriptive design describes people’s life experiences regarding a particular phenomenon [25]. Qualitative descriptive design allowed the researchers in this study to understand contributing factors to unsafe abortion practices by women in the Ashanti Region of Ghana.

### Study area and population

This study was conducted in four selected district hospitals in the Ashanti Region of Ghana. There are 25 district hospitals located in the Ashanti Region [26]. Most of these hospitals had gynaecological units that treated women for unsafe abortions. However, most of these facilities do not have institutionalised systems for the implementation of Ghana’s abortion protocols and policies on safe abortions [27]. The Ashanti region is the second highest (20.8%) in Ghana with women who have ever attempted unsafe abortions [15]. The study population were patients, religious leaders, midwives and medical officers within the ages of 15 and 65. All patient participants were within the ages of 15 and 49 and were being treated for unsafe abortion complications at selected district hospitals in the Ashanti Region. Table 1 shows the number of midwives and medical officers in gynaecological units at selected sites of study. The table (Table 1) also shows the number of unsafe abortion patients over a period of 3 months at selected sites of study.

**Table 1:** represents Number of midwives, doctors and patients by hospital

Hospital	Number of Midwives in gynaecological units	Number of doctors in gynaecological units	Number of unsafe abortion patients in 3 months
Site 1	20	5	35
Site 2	25	6	30
Site 3	23	7	20
Site 4	26	4	18
Total	94	22	103

### Data collection

Purposive sampling technique was used to recruit one hundred and eleven (111) participants (Table 1). The sample size was determined by qualitative data saturation.

Thirty-five (35) of the one hundred and eleven (111) participants were patients between the ages of 15 to 49. A total of one hundred and three (103) patients were treated for unsafe abortions complications in the four selected hospitals during the period of data collection (Table 1). Inclusion criteria for patient participants was individuals who had self -induced or sought services of untrained personnel to terminate their pregnancies and were being treated for complications of unsafe abortions. Patients’ folders were read for the history of presenting complaints and confirmed by patients themselves as people who indulged in unsafe abortion practices for which they were hospitalised. Selected patients were involved in semi-structured interviews.

Thirty-five (35) of the one hundred and eleven (111) participants were Christian and Islamic religious leaders. These religious leaders lived in districts where hospital sites were located. Religious leaders were involved in seven focus group discussions (FGDs) where each focus group included five Christian and Moslem leaders. Religious leaders were mostly pastors and Imams. However, other members of churches and mosques who were identified by pastors or Imams as performing leadership roles were included.

Thirty (30) of the one hundred and eleven (111) participants were Registered Midwives who were practising in the selected hospitals in the Ashanti region. These midwives were involved in 6 FGDs where each group was made up of 5 midwives.

Eleven (11) of the one hundred and eleven (111) participants were medical officers who were practicing at Gynecological wards of selected hospitals in the Ashanti Region. These medical officers were involved in semi-structured interviews.

Only individuals who could speak either English or Twi (a local language spoken by residents of Ashanti Region) were included as participants. All data were collected through key informant interviews and FGDs. Interview and FGD questions were developed by the research team that included two nursing education experts. Questions asked included the following (Additional file 1): 1. Describe your knowledge of safe abortion services in your community or country. 2. Describe situations or circumstances that made you practice unsafe abortion. 3. Describe your religious and cultural beliefs about abortion. 4. Describe the reasons that prevented you from keeping your pregnancy. 5. Describe any impact of your pregnancy on your life for which you decided to have an unsafe abortion. 6. Describe any impact/influence of relationships with friends and family/society on your decision to have an unsafe abortion. 7. Describe any other social or economic issues that influenced your decision to have an unsafe abortion. Probes were used to elicit further descriptions of factors that influenced practices of unsafe abortions. All questions can be found in Additional file 1. Data collection instrument was translated into Twi by a language expert for participants who could not speak English. The interview guide was pretested on five patients, three midwives, three medical officers and five religious leaders in a similar district. The results of the pretest were used to modify questions for clarity. All ambiguous questions were either modified or removed. Data were collected from January to March 2018. Interviews and FGDs lasted between two and 4 hours. Data saturation was reached after interviewing 46 participants and involving 65 participants in FGDs. Transcribed data were stored with a password. Folders containing data transcriptions were kept on a pen drive solely meant for the purposes of the study and kept under lock and key.

### Data analysis

Holloway and Wheeler’s [28] data analysis pattern was used during data analysis. This pattern takes the following form; validating, transcribing, cleaning and coding data. The researchers employed the following activities: transcription, validation, cleaning, and coding. Themes were developed through content analysis of data collected. The team of researchers who collected came together to transcribe data from audio recordings. Transcripts were read several times by the team to identify codes. The team used similar code to create families and similar families grouped together as themes. Themes were discussed by all researchers to make sure they reflected the phenomenon that was captured during data collection. Some participants were also consulted to make sure the themes developed represented their views. Participants were identified with alphabets and numbers in order to maintain anonymity: PP11 (patient participant number 11), FGR37 (focus group religious leader 37), FGM3 (focus group midwife number 3), MO2 (medical officer number 2).

### Rigour

An interview guide was pretested on five patients, three midwives, three medical officers and five religious leaders. The pre-test helped researchers to modify questions for clarity. The pre-test also helped to ensure that the data collected answered the research questions. The research team had prolonged interactions with the participants to ensure an in-depth understanding of emerging findings. Data transcriptions and coding were done by the research team to ensure that the correct experiences and views of the participants were reported. Discussion of themes by the research team was done to ensure correct representations of participants. Study participants were consulted for their comments on themes to make sure it represented their views.

### Ethics approval and consent to participate

The study was approved by the Institutional Review Boards of Kwame Nkrumah University of Science and Technology. Administrative approval was received from district hospitals where the study was conducted. The study was explained to participants. Anonymity and confidentiality were explained to participants and they were assured that withdrawal will not in any way attract sanctions. Consent forms were signed by participants after necessary explanations of consent procedures. Participants were identified with codes to maintain anonymity and confidentiality. The study did not cause any physical or psychological harm to any participant. Consent to publish was also granted by participants.

## Results

### Thematic results

Seven thematic categories were extracted from data: a) Lack of knowledge of safe abortion services; b) Socio-economic conditions as perceived influence for unsafe abortion practices; c) Safe abortion as a perceived religious and cultural taboo in Ghana; d) Stigma of unplanned pregnancy; e) A desire to bear children only after marriage; f) Avoiding parental/guardian disappointment and resentment; g) A desire to pursue education.

### Lack of knowledge of safe abortion services

Majority of participants demonstrated poor knowledge of safe abortion policy and services in Ghana. As many as 90 participants that included patients, nurses, religious leaders and medical officers indicated that they did not know of the safe abortion policy and services in Ghana. Lack of knowledge on safe abortion services led to unsafe abortion practices by women who had unplanned pregnancies. Though almost all patient participants interviewed could identify complications of unsafe abortion practices such as bleeding, death, uterine damage, infertility, gastric damage, and infections, many said they indulged in unsafe abortion practices because they did not know of the safe abortion option. This phenomenon was expressed in the following statements by participants:

*I didn’t know where to go and terminate the pregnancy. I knew that it is illegal to have an abortion in Ghana and so I could not have gone to any facility to have by pregnancy terminated. All my friends that I asked only recommended some herbal mixture called agbeve for me. They also did not know of any hospital where I could boldly go and abort safely. I read on the label that agbeve is not to be taken by pregnant women. When I read that I had hope that it will get rid of my pregnancy. Although I know I could bleed to death from terminating my own pregnancy, I didn’t have a choice or options. So I used the agbeve herbal mixture [PP11].*

*I know abortions are illegal in Ghana so I couldn’t go to any hospital looking for a safe abortion. I did not want to be caught doing an illegal thing such as abortion which people see as murder here. I could not also keep the pregnancy because it will be a disgrace to me and my parents. No one has ever informed me of safe abortion services in any part of Ghana. I wish that existed and I will not have to try aborting my pregnancy myself. I have heard that some women have become infertile. I had to use aunty mercy herbal mixture to terminate my pregnancy. Unfortunately I stated bleeding plenty and a friend brought me here [PP22].*

*Well I am a registered midwife here and no one has ever taught me that abortion in Ghana is legal. In fact what I know is that it is illegal to have an abortion in Ghana. I feel that doctors who perform abortions are undertaking illegal activities so I don’t even feel like helping them. They normally charge the women lots of money to perform the abortions. I think they are taking the risk because of the money they get from these clients. The patients sometimes tell you the truth of the kind of drug they tried using. Many of them have used the agbeve herbal mixture. Agbeve is not supposed to be taken by pregnant women but instead they take it to abort their pregnancies [FGM3].*

*I only know about family planning services but not abortion services. As for abortion it is a sin. Every life is precious to God. We are not supposed to take life in a form of abortion. In psalms, God said the human beings are his people and he cares for us. It is not humans who should be taking lives. Even in Isaiah, God showed us that he cares for even unborn children so no one is supposed to take the life of these babies. In Jeremiah God made it clear that before we were born he knew us and cared about us. And I know our constitution is based on the bible so this country and its constitution will never allow for abortion in our land [FGR37].*

*Abortion is not allowed in Ghana. I have heard there is some law that permits abortion under some circumstances. As for abortion policy, I don’t know of any. People see abortion as a sin here. I do too. Usually our view in Ghana is that it is murder of babies. These are the reasons why young women who get pregnant feel they have to find other means of terminating their pregnancies rather than coming to us for help [MO 9].*

### Socio-economic conditions as perceived influence for unsafe abortion practices

Many participants who were involved in unsafe abortion practices admitted that their socio-economic conditions made them indulge in unsafe abortion practices. Respondents cited financial difficulties, schooling, and non-readiness to cater for a baby as reasons for practising unsafe abortions. Socio-economic conditions as a contributing factor to unsafe abortion practices are described in the following statements by participants:

*I can’t look after a baby if I carry it to term. I took a home pregnancy test and it came out positive. I am not with the guy nor do I want to be with him. I considered an abortion because I could not afford to carry the pregnancy to term. I don’t want to be a single mother because I don’t have the money to look after myself and a child at this time of my life. I did not hesitate to abort it by taking concoction of aunty mercy herbal drug which was suggested by a friend [PP8].*

*There was no question of me keeping the pregnancy because I knew I was going to be admitted to the university. I would have a good education and I had a career path to go down. It was all laid down for me. I couldn’t sacrifice my schooling for motherhood. I could not combine the two because I don’t have the money to do that now. I am not ready for motherhood. I do not think lack of money is a good reason to have an abortion but the reality is that you can’t take care of a baby without a job and money so I found a way to abort the baby by myself [PP12].*

*I have seen many patients who have reported here when they have already attempted to abort their pregnancies and had complications. I think they fear the cost that they will have to bear when they come here, apart from the criminal aspect of it. Apart from being afraid that they are doing criminal things, and stigma, money is an issue for these young people who get pregnant and want their pregnancies terminated [FGM134].*

*Normally when they come like that, you can see that they don’t have money to buy the medications that we need to use to help them. Even the procedure to completely remove the remains of what they started, some of them find it difficult to pay for it. As for this one the national health insurance will not pay so they have to pay themselves [MO7].*

### Safe abortion as a perceived religious and cultural taboo in Ghana

Majority participants (89 out of 111) indicated that safe abortion of a pregnancy is deemed a sacrilegious act in the Ghanaian society. All participants were Christians and Muslims who were against abortion services. Participants were against abortions in any form, whether safe or unsafe. Participants expressed their religious abhorrence of abortions in the following statements:

*Abortion is not permitted in my religion because it is an offence against God. It is the killing of a human being which can even lead you to jail. I had an unsafe abortion by myself and I regret it more than ever and get depressed about it anytime I think about it. Abortion is bad because my religious doctrines speak against it. Abortion is the same as killing. It is a killing of a living breathing baby that God created. Life is not ours to give and take away. I feel God will not forgive me for what I have done [PP 7].*

*I am a Muslim and I can tell you that we don’t agree to abortions. Taking the life of an unborn child is a sin against God and man and when one commits abortion the person kills which is against our beliefs. My religion completely frowns on the practice of all kinds of abortions [FGR1].*

*I am a Christian and abortion whether safe or unsafe is seen as murder. We are not even supposed to get pregnant out of wedlock. It is a sin to have sex with someone you are not married to. We preach against these things all the time. People must change and ask for forgiveness otherwise they are lost souls [FGR6].*

*Abortion is not allowed here. It is a sin. Personally my religious beliefs don’t allow for me to just abort a pregnancy for a client. I stay away from such acts. I only get involved when it is a life and death issue. When the patient has already attempted and is in danger of dying, that one I have no choice but to help save life. People feel why do you take a life that you didn’t create? And I think it is a legitimate question. All Christians and Muslims in this community see it as religiously wrong. It is just not acceptable [MO 4].*

*Religions in Ghana don’t support abortion. We have two main religions in Ghana and I know that none support abortion. I don’t support it because of my religion. No good Christian or Muslim will allow for abortion of an unborn baby [FGM 12]*

Many participants (99 out of 111) in this study also indicated that abortion is against Ashanti and Ghanaian culture. Many participants stated that they practised unsafe abortions because their society did not approve abortion even if it was a safe one. Participants were afraid their families and friends would be disappointed and call them names if they found out that they were pregnant and wanted to abort their babies. The cultural detestation was expressed in the following statements by participants:

*Our culture here is very much against abortion. I don’t think anyone here agrees with people having abortions. It is seen as killing so people do not condone it at all here. If people get to know you aborted a baby, they will always be pointing fingers towards you when you are passing. So you have to find a way of doing it without people knowing, to avoid the stigma [PP33].*

*Our community perceives abortion to be a bad practice and anyone who engages herself in it suffers as a result of people stigmatising her. This is because it brings disgrace to victim’s family and the whole community. My society and culture see abortion as very criminal, unacceptable and an abomination. They see it as a taboo. It brings disgrace to the family and if care is not taken it can lead to embarrassment for the woman and people around her [MO5].*

*Sexual intercourse is only legitimate in marriages. Therefore, when a woman gets pregnant without a husband, it becomes a disgrace to the woman. Apart from religions reasons for not allowing abortions, the culture here does not allow abortions. The whole society here is against abortion [FGR57].*

*As a nurse I know our culture does not support abortion. We see it as killing in Ghana. I am sure anyone you talk to in Ghana will tell you that abortion is not good. To be honest with you, it is always seen as killing [FGM11]*

### Stigma of unplanned pregnancy

Many participants (95 out of 111) said pregnancy before marriage is not acceptable in the Ghanaian society. Therefore, women who got pregnant before marriage were most often stigmatised in their community. Many people in the Ashanti region expected women to get engaged and properly married before getting pregnant. Women who became pregnant outside wedlock avoided embarrassment by aborting their babies through unsafe means. Patients, religious leaders, midwives and medical officers stated that stigma of unplanned pregnancies was a major reason for unsafe abortions in Ghanaian communities. The stigma of unplanned pregnancies resulting from the unacceptability of pregnancy outside marriage is expressed in the following statements by participants:

*The society perceives pregnancy outside wedlock as fornication because the person is not being married but has given birth from nowhere. Sexual intercourse is only legitimate in marriages. Therefore, when a woman gets pregnant without a husband to cater for the unborn child, it is a disgrace to the woman and her family. This forces the woman to abort the pregnancy in order to avoid disgrace and social stigma. In my case I was thinking about what people will think of me knowing how my society is [PP7].*

*Pregnancy outside wedlock in my society is perceived as fornication. The child when it is born becomes a bastard. The woman who gets married before having babies is always admired but the person who gets pregnant without marriage is seen as a spoilt girl. Though no civilized society permits one human to intentionally harm or take the life of another human through abortions, women sometimes have no choice because of the embarrassment unplanned pregnancy brings. The stigma is serious here [FGM33].*

*It is nice to attend engagement and wedding ceremonies where women get married before they get pregnant and have babies. That is what is accepted in this community. Anything aside this is a disgrace to the woman and her family. Apart from being religiously wrong, the society does not accept such behaviours. We don’t want our young women getting pregnant without marrying. If they are not ready for marriage, they should not be doing what marriage people are supposed to be doing. Sex is for only married people. For me when I see a girl pregnant without marriage, I see her as a bad girl [FGR12].*

*We usually see people who get pregnant outside wedlock as prostitutes. You have to marry before you get pregnant and have babies. Some young women these days get pregnant without getting married and our society does not respect them at all. The way people will be looking at the pregnant girl, she herself will feel very embarrassed. They normally regret these pregnancies and so attempt to abort them [MO1].*

### A desire to bear children only after marriage

Majority of participants in this study (98 out of 111) indicated that they desired marriage before bearing children. This desire is brought about by the admiration and respect offered individuals who get married before bearing children. Though some women get pregnant without getting married, they usually believe their chances of getting someone to marry is higher if they get rid of their unplanned pregnancies. This phenomenon is seen through the following statements by participants:

*In this community, it is a pride for a woman to have a man marry her in a nice ceremony and be recognised as officially married. Every woman wants such an honour and will even prefer aborting an unplanned pregnancy so that someone can marry her. My parents have always wanted me to get married to a man before getting to have babies so I was embarrassed when I found out that I was pregnant. It is difficult to get a good man to marry you when you are a born one. People think you are already used. I talked to a trusted friend and she led me to a place where someone tried to abort the baby for me. It was however not successful because I started having severe abdominal pain and passed out and had to be brought here [PP31]*

*It is an honour for women to get married in Ghana. Marriage ceremonies here are more or less a ceremony to glorify women in our society. Most women would want to be married before they have children so they can enjoy that respect and honour given to other women who have done that. Sometimes when you talk to the young women who attempt abortions and are brought here, they tell you that their friends, and families will stop respecting them when they have babies out of wedlock. All of them want marriage before bearing children [FGM30].*

*Everyone woman here will always wish that she gets married before having children because of the respect for women who do that. Your dignity as a woman is seen when you marry before getting pregnant. That is how it is here. Many women will prefer aborting on their own to avoid the embarrassment of having babies without a husband [MO9].*

*Our religious inclinations tell us that people must marry before having babies. Right from the era of Adam and Eve, it has been shown that men and women cannot just start having sex and having babies without officially getting the recognition as husband and wives. It is not acceptable. Whether you are Muslim or Christian you will need to do the official thing before having children. I think this is part of the reason why some of our women will prefer to avoid the embarrassment by taking away the life of their unborn children. That one too is against God’s rules [FGR28].*

### Avoiding parental disappointment and resentment

Many participants (80 out of 111) indicated that many pregnant young women would always want to prevent disappointment and resentment from parents/guardians about their unplanned pregnancies. They would want to maintain existing cordial relationships between them and their parents/guardians as many of them were still living under the care of their parents or guardians. The attempt to maintain cordial relationships with parents/guardians and avoid disappointments and resentment from them is seen in the following statements from participants:

*I didn’t know what my parents will think of me getting pregnant whilst in school. My mum for instance has always talked to me about the need to remain a virgin until I get married. In fact she believes I am still a virgin so I didn’t want her disappointment about the pregnancy so I had to take steps to do something about it. She will always tell me finish school, have a good job and marry and have beautiful babies. She would have even gotten angry with me. I was scared about the reaction of both my Dad and Mum [PP14].*

*I am still in school and being looked after by my guardian. My parents brought me to the city to stay with my aunty who is looking after me in school and in everything. My aunty expects me to finish school and get married before thinking about babies. I cannot all of a sudden tell her that I am pregnant. She may get angry and take me back to the village or ask me to leave the house. To avoid all these I just had to find a way of terminating my pregnancy since I had no other option. My boyfriend too is a student and does not have the money to look after me and the child now [PP17].*

*When you take history of illness from these young women, you realise that their parents were not aware of their attempts to terminate their pregnancies. They always want to hide it from their parents because of the embarrassment, disappointment or anger from parents or guardians. They will always prefer confiding in friends when they want to terminate their pregnancies rather than talking to their parents [MO10].*

*Honestly the ladies who attempt termination of pregnancies before coming here most often than not don’t tell their parents. Sometimes it is even their friends that bring them here. May be they don’t feel comfortable talking to their parents about it. May be to avoid any ill feelings from parents they would rather tell their trusted friends. They probably fear the disgrace and disappointment from parents who are still looking after them [FGM3].*

*They will always feel embarrassed by getting pregnant because it not part of our culture or religion to have women getting pregnant before marriage. Most of these girls are still in school and still get pregnant. It is not good enough. Their parents should definitely feel disappointed. I will feel same way too. I think they need more education. As for me when I hear and see these things I feel bad within my spirit [FGR5].*

### A desire to pursue education

Many participants (97 out of 111) in this study stated a desire to pursue education as a factor that leads to unsafe abortions in Ghanaian communities. Almost all patient participants were at various levels of their education and did not want to truncate their education as a result of pregnancies. Religious leaders, midwives and medical officers also thought the desire to continue with education made some students attempt terminating their pregnancies. This theme is evident in the following statements by participants:

*I have always wanted to finish school and be on my own and also work as my parents do. When I realised I was pregnant I thought about my schooling and I could not afford to sacrifice my education for family life as a single mother. The future looked bleak without my education. So I had to talk to by boyfriend who bought some herbal medicine for me. Unfortunately it didn’t work well [PP21].*

*I am a students in the university. My whole future is ahead of me which can only be better with education. I can’t sacrifice that for a pregnancy whose father isn’t ready either. So I am left with no better decisions than try aborting this pregnancy. Hopefully I will finish school and get a job and live a better life [PP7].*

*The patients that we see are mostly students either in senior secondary or in their tertiary education. They usually say that when they carry their pregnancy to term and deliver, it will disturb their education. They cannot imagine studying with pregnancies. Some also fear that they will be sacked from school because of the pregnancy [FGM19].*

*The patients we have seen here are people who want to continue being in school and don’t want their pregnancies to make them stop schooling. In Ghana, some schools actually sack their students from school. These students see their colleagues being sacked especially from the junior high and senior high schools and so they don’t want to go through the same ordeal. I think now the ministry says school administrators should not sack their female pupils or students but I think it is still happening in some places. Normally they say they try using agbeve herbal tonic to abort so they can be free to continue their schooling [MO3].*

*In this country, you are mostly withdrawn from school when you get pregnant. That is why they have to concentrate on their schooling and not getting pregnant. How can you be in school and get pregnant whilst your parents are trying to do their best to look after you? It is not the best. I have actually heard that some head teachers sack them from school [FGR10].*

## Discussion

This study assessed contributing factors to unsafe abortion practices among women in the Ashanti Region of Ghana. Contributing factors to unsafe abortion practices are discussed in line with the seven thematic categories that were extracted from data.

### Lack of knowledge of safe abortion services

**Fig. 1:**
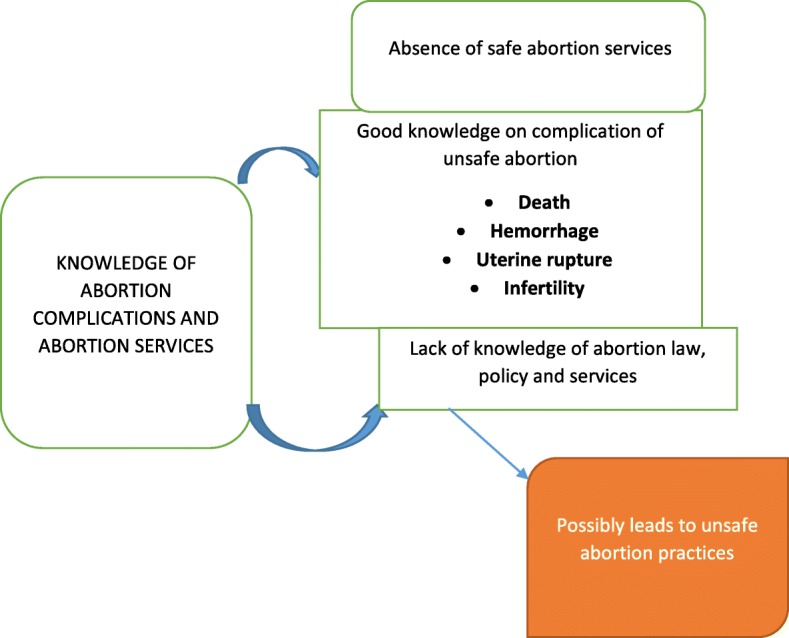
Poor knowledge of abortion law and safe abortion services as a contributing factor to unsafe abortion practices. Source: conceptualised by authors (2018). This figure represents the first thematic result of this study with heading ‘Lack of knowledge of safe abortion services’

Majority of patient participants were Christians, single and unemployed (Table 2). Many of them were in secondary schools, colleges and universities (Table 2). Many women in this study engaged in unsafe abortion practices because they lacked knowledge on abortion policies and services in Ghana (Fig. 1). Despite the fact that there is an existing reproductive health policy in Ghana which specifies the need for safe abortion services in Ghanaian health facilities, many health tutors and health providers lack knowledge on the policy [18, 19]. A study by Voetagbe et al. [29] found that a high proportion of midwifery tutors did not know of the policy governing the provision of safe abortion care in Ghana. It is not surprising that many health providers particularly nurses do not educate women on abortion services in Ghanaian health facilities as part of their reproductive health care for women. According to Sedgh [9], knowledge of the country’s moderately liberal abortion law is not widespread among health professionals. Lack of knowledge on the legality of abortion services transcends to the general public especially women who are supposed to be aware and take advantage of these services. A Ghana Maternal Health Survey found that, in 2007, only 4% of women knew that safe abortion was legal in Ghana [27]. Most women still regard safe abortion practice as illegal in Ghana and this usually results in unsafe abortion practices by these women (Fig. 1). Some legal practitioners have proposed more amendments to the abortion law to enhance the implementation of safe abortion services in Ghana [30]. This is because over the years the reproductive health policy of Ghana only dwelt on the promotion of family planning, contraception and post-abortion care but not a provision of safe abortion within the confines of the law as recommended by World Health Organization (WHO) [29, 30]. Education and policy implementation of the safe abortion policy and services in all health facilities in Ghana is crucial since results in this study shows inadequate knowledge of abortion policy and laws leads to unsafe abortion practices (Fig. 1). There is the need for strengthening of adolescent reproductive health education programmes at secondary and tertiary levels. There is an urgent need for national education on the abortion law and policy by the National Commission on Civic Education (NCCE). If teachers are not sure of the law, the midwives they train will also likely be uncertain of the conditions under which they are legally allowed to provide safe abortion services [31]. The curriculum of health professionals must incorporate the positive aspects of the abortion law in Ghana. Continuous professional development programs should be organised for health professionals to enlighten them the on the progressively liberal abortion law of Ghana. Health professionals must realise that abortion is a fundamental human behaviour that has been practiced in all cultural settings and that no level of restrictive laws has succeeded in controlling it [32]. Thus when a woman decides to end an unwanted pregnancy she will often go to extreme length to do so, regardless of whether the procedure is safe or legal and as long as there are unwanted pregnancies, abortion will be a fact of life [20]. Other teachers in senior high schools, colleges and universities should also be involved in the education on safe abortion services in Ghana so that they can in turn educate students. Moving beyond doctors to involve a wider range of health workers is an increasingly important public health strategy [33]. Planned and regulated task shifting and task sharing can ensure a rational optimisation of the available health workforce, address health system shortages of specialised health-care professionals, improve equity in access to health care and increase the acceptability of health services for those receiving them [33]. Nurses and midwives in Ghana must, therefore, have the necessary knowledge in order to transmit same to the general public especially women within the reproductive age group. Though evidence available shows that self-management of abortions with the use of abortions pills (misoprostol alone or misoprostol in combination with mifepristone) can reduce maternal mortality and morbidity, inadequate information can lead to wrong dosages which will subsequently end in unsafe abortions [1]. The regimen for medical abortion needs to be publicised by the Ghana Health Service (GHS) but with the appropriate education. There are very few websites that support women by answering pertinent questions about abortions [34]. Women Help Women which is an international organization is contributing its quota by launching a service called Self-managed Abortion, Safe and Supported (SASS) for women all over the world [34]. Ghanaian women can also benefit from SASS if it is given the necessary publicity. The SASS website is managed by counsellors who answer questions through secure emails [34]. Access to safe abortion depends on the availability of trained midwives and medical officers [35]. The American College of Obstetricians and Gynecologists supports an increase in the number and types of trained professionals that can lead to improved accessibility of women to safe abortions [35]. An example of effective methods of transmitting safe abortion information to communities is that of Aunty Jane (Malawi and Kenya) and Ms. Rose (Nigeria) where safe abortion information telephone hotlines are constantly available to answer questions on abortion [36]. These public health strategies can be adopted in Ghana to reduce maternal mortalities and morbidity that occur as a result of unsafe abortion practices.

**Table 2:** This table shows the summary of the descriptive statistics (frequencies and percentages) of the demographic characteristics of respondents

Variable	Patients	Religious leaders	Midwives	Medical officers
	N (%)	N (%)	N (%)	N (%)
Gender				
Male	–	33 (29.72)	–	8 (7.2%)
Female	35 (31.5%)	2 (1.8%)	30 (27%)	3 (2.7%)
Age				
15–25	20 (18%)	3 (2.7%)	2 (1.8%)	–
26–35	10 (9%)	6 (5.4%)	20 (18%)	4 (3.6%)
36–45	4 (3.6%)	10 (9%)	3 (2.7%)	4 (3.6%)
46–55	1 (0.9%)	10 (9%)	3 (2.7%)	2 (1.8%)
56–65	–	6 (5.4%)	2 (1.8%)	1 (0.9%)
Religion				
Christianity	25 (22.52)	20 (18%)	25 (22.5%)	8 (7.2%)
Islam	10 (9%)	15 (13.5%)	5 (4.5%)	3 (2.7%)
Marital Status				
Single	33 (29.72)	9 (8.1%)	15 (13.51)	8 (7.2%)
Married	–	24 (21.62)	14 (12.61)	3 (2.7%)
Divorced	2 (1.8%)	2 (1.8)	1 (0.9%)	–
Educational Level				
No formal education	5 (4.5%)	–		
Primary school	5 (4.5%)	5 (4.5%)	–	–
Secondary school	10 (0.9%)	15 (13.51)	–	–
Tertiary level education	15 (13.51)	15 (13.51)	30 (27%)	11 (9.9%)
Employment				
Employed	2 (1.8%)	35 (31.5%)	–	11 (9.9%)
Unemployed	33 (29.72)	–	30 (27%)	–

### Socio-economic conditions influence unsafe abortion practices

**Fig. 2:**
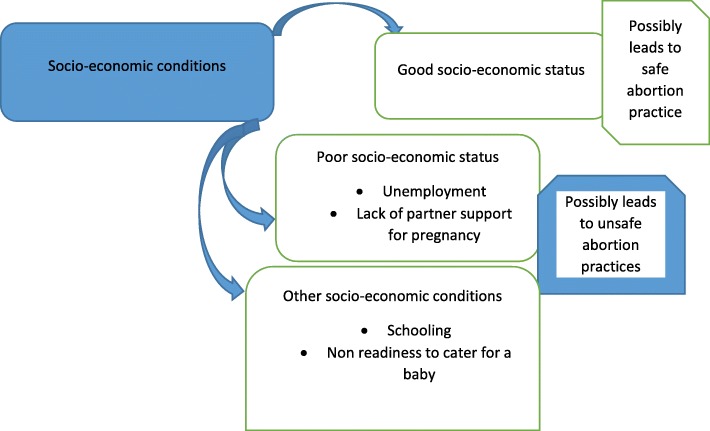
Socio-economic conditions as contributing factors to unsafe abortion practices. Source: conceptualised by authors (2018). This figure represents the second thematic result of this study with the heading ‘Socio-economic conditions influence unsafe abortion practices’

Many participants in this study admitted that their socio-economic conditions such as financial difficulties, unemployment and inadequate economic support made them indulge in unsafe abortion practices (Fig. 2). Many of these women were also in school and were not ready to be mothers (Fig. 2). Women who are most vulnerable to unsafe abortions are usually younger, poorer, and lack partner support [37–41]. Safe abortion has frequently become the privilege of the rich, while poor women have to resort to unsafe providers, causing deaths and morbidities that become the social and financial responsibility of the public health system [28]. Some Ghanaian legal practitioners believe the law on abortion needs more amendments to improve clarity to all stakeholders [20, 27]. Further amendments to the abortion law in Ghana will remove current bottlenecks where stakeholders such as health professionals and women believe safe abortion services are illegal and therefore need to pay more for it as a service in formal health facilities. A liberal interpretation of the law must be encouraged among stakeholders of the health sector in Ghana. The current safe abortion policy should be publicised to prevent clandestine abortion services that result in maternal morbidity and mortality. Majority of women who had unsafe abortions were found in this study to be below age 25 of which most were in tertiary and senior high schools (Table 2). In a study conducted in Ghanaian universities, 91% of students called for the establishment of a reproductive health counselling centre on their campuses [40]. Sexual and reproductive health services are needed to help women avoid unplanned pregnancies, and to ensure healthy outcomes for those who do experience such pregnancies [42]. On-campus reproductive health services can be provided to students in senior high schools, colleges and universities in Ghana at a reduced or subsidised cost. This reproductive health services should include sex and family planning education. These on-campus reproductive health services should also include education on the safe abortion policy and services in Ghana.

### Safe abortion as a perceived religious and cultural taboo in Ghana

**Fig. 3:**
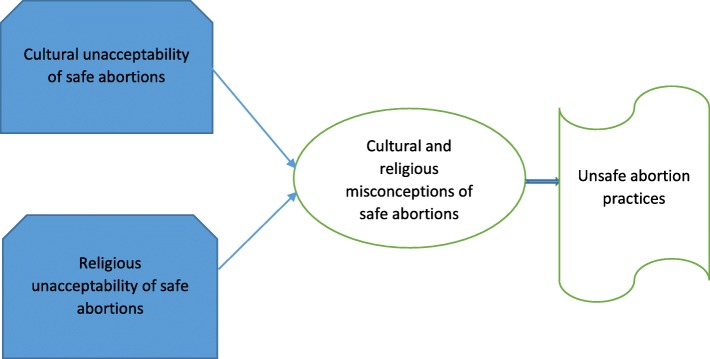
The cultural and religious unacceptability of safe abortion as contributing factor to unsafe abortion practices. Source: conceptualised by authors (2018). This figure represents the third thematic result of this study with heading ‘Safe abortion as a religious and cultural taboo in Ghana’

Participants in this research indicated that they indulged in unsafe abortion practices because safe abortion of a pregnancy is deemed a sacrilegious religious and cultural activity in Ghana (Fig. 3). Christianity, Islam and traditional religions are the three main religions in Ghana and almost everyone belongs to one of these religions [43]. Religious leaders (pastors and Imams) who were interviewed in this study indicated that the Bible and Quran were against abortions. There are debates about whether the moral behaviours of the Ghanaian citizen is influenced by religion rather than society and traditions. Some scholars believe that the moral beliefs of the Ghanaian are religiously determined whilst other hold the contrary view [43]. A recent study by Anderson argued that Ghanaian society’s beliefs are currently determined by religions because all cultures are affiliated to the various religions in Ghana [43]. Some researchers have concluded that replacement of Ghanaian cultural practices that prevented immoral sexual activities with foreign cultures is leading to increased unwed pregnancies and abortions [44]. Earlier studies found that many Ghanaian nurses and medical officers feel providing abortion services was in conflicts with their religious and cultural beliefs [45]. Lack of knowledge of the Ghanaian abortion law and services, coupled with cultural and religious stigma results in clandestine procedures from untrained providers or self-induction [17, 45]. An impact assessment of a training programme on the Ghanaian abortion law in the Eastern and Greater Accra Regions found that negative attitudes toward safe abortion services remained almost the same even after training [23]. This negative attitudes may be due to socio-cultural and religious views that impede the provision of safe abortion services. Sub-Saharan Africa women’s access to safe abortion care is hampered by socio-cultural barriers [8, 27, 46–49]. The WHO found that lack of social support and providers’ negative attitudes were barriers to safe abortion in less developed countries [30]. Culturally, abortion is a taboo topic among many tribes in Ghana [34, 49]. It is seen as an embarrassing and shameful act that is practiced by immoral women [27, 47, 50]. This makes abortion a stigmatised practice in Ghanaian communities [27]. Researchers have also established that individual religiosity influences abortion attitudes, and that abortion attitudes, in turn, shape abortion restrictions and access where most opponents cite religious reasons for their opposition [51–53]. It is imperative to increase education on the benefits of safe abortion services. It is unacceptable to turn a blind eye to the death of women due to the social, cultural and religious stigmatisation of safe abortion practices in Ghana. The issue of forgiveness for safe abortion should not be decided by the general populace but should be left to the deity that the individual persons worship. The laws and policies regarding safe abortion services must be implemented regardless of the cultural and religious attitudes towards the availability of such services.

### Stigma of unplanned pregnancy

**Fig. 4:**
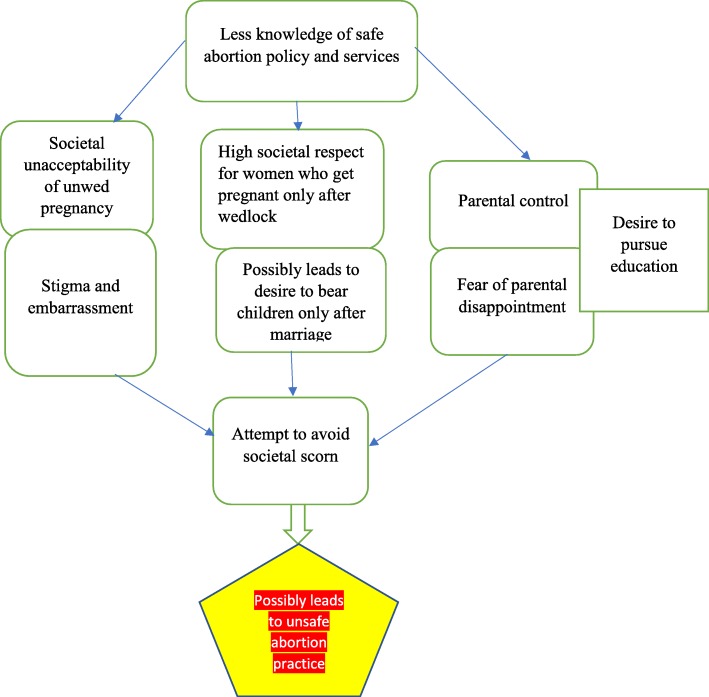
Societal unacceptability of unwed pregnancy, desire to bear children only after marriage, and fear of parental disappointment possibly leads to unsafe abortion practices to avoid societal scorn. Source: conceptualised by authors (2018). This figure represents the 4th, 5th and 6th thematic result of this study with headings ‘Stigma of unplanned pregnancy’, Desire to bear children only after marriage’, Avoiding parental disappointment and resentment’ and ‘Desire to pursue education’

Patient participants indicated that they indulged in unsafe abortions because they feared the stigma that accompanied unwed pregnancy in Ghana (Fig. 4). Religious leaders, midwives and medical officers indicated that pregnancy before marriage is unacceptable in Ghanaian society. Therefore young women who got pregnant before marriage decided to terminate their pregnancies to avoid the stigma that comes with getting pregnant without marriage. The unacceptable perceptions towards out of wedlock pregnancy and abortions in Ghana may result from religious affiliations of Ghanaians. Majority of Ghanaians are Muslims and Christians. Moral beliefs influences laws and policies in many countries [54]. Majority of religious leaders interviewed in this study stated that both unwed pregnancy and abortions were unacceptable in Christian or Muslim religions. Many patient participants said they had to use unsafe and unapproved means of aborting their pregnancies because of the fear of stigma that resulted from pregnancy out of wedlock. Guttmacher Institute and the United Nations Population Fund (UNFPA) estimated the level of unplanned pregnancies at 49 per 1000 pregnancies in Asia, 72 per 1000 in Latin America and the Caribbean and 86 per 1000 in Africa [55]. Unplanned pregnancy rate in Africa was rated as the highest [51]. Guttmacher Institute and the United Nations Population Fund (UNFPA) also stated that in Ghana, 37% of all births are unplanned [55]. In Ghana, marriage before pregnancy is a cultural and religious expectation of adult women. Pregnancy before marriage is seen as a dishonour to womanhood. As a result of the cultural and religious expectations of marriage before pregnancy, many unmarried women who get pregnant prefer to abort their babies to avoid any public spectacle and scorn. Many researchers have found that unplanned pregnancies carry serious consequences such as stigma for women and their families leading to unsafe abortions [56–58]. The rate of unplanned pregnancies in Ghana shows inadequate accessibility of reproductive health services by women [59]. The solution to this challenge could be increased health education on contraception which could help prevent unplanned pregnancy and the consequent stigma that comes with it. Additionally, stakeholders such as professional bodies, the ministry of health, Ghana Health Service and health providers must endeavour to implement the law and policies of safe abortion in order to prevent deaths among women due to unsafe abortion practices. Three major strategies that would help to destigmatise abortion in the Ghanaian community and allow women with low socio-economic status access to safe abortion services at an affordable cost has been suggested. These strategies are (1) the liberal interpretation of the law on abortion; (2) expanding community awareness of reproductive health benefits of safe abortion services; and (3) improving and increasing access to legal abortion services within health facilities in Ghana [27].

### Desire to bear children only after marriage

Majority of participants stated they had unsafe abortions because they desired to bear children only after marriage (Fig. 4). Though authors in this study did not find any related study to this theme, having a biological child is an essential part of marriage in Ghana [60]. The pride of having children only after marriage is a predominating phenomenon in Ghana. Some studies have found that advantages exists for children who are born in intact families rather than non-intact families such as co-habitating and single parenthood [61]. On many social, educational, and psychological outcomes, children in cohabiting or single households perform significantly worse than children in intact, married families [61]. Despite the desire to bear children only after marriage, a study in the United State of America found that the percentage of live births that were born to unmarried women had increased from 5.3% in 1960 to 72.3% in 2011 whilst percentage of children under age 18 living with two married parents has decreased from 88% in 1960 to 65% in 2011 [61]. More children are being born out of wedlock. Ghanaian women prefer to bear children after marriage because of the social and psychological advantages that currently exist for doing so. Marriage occurs relatively early in Ghana, and one in four women age 20–24 are currently married [62]. Marriage marks the point in a woman’s life when childbearing becomes socially acceptable in Ghana [62]. In Ghana, by age 18, more than two-fifths of women (44%) and 26% of men have had sexual intercourse. Though more women than men have sexual intercourse by their 18th birthday, knowledge on contraceptives is higher in men than women [62]. Unless strategies are developed to increase knowledge of young women on contraceptives, the desire to bear children only after marriage will only be achieved through abortion of unplanned pregnancies. In the absence of safe abortion options, these young women will opt for unsafe abortions methods of getting rid of their unplanned pregnancies thereby increasing maternal morbidity and mortality.

### Avoiding parental disappointment and resentment

Findings in this study show that young women had unplanned pregnancies because they feared the disappointments and resentments of their parents or guardians. Unsafe abortions were practiced to avoid the disappointment and resentment from parents and guardians (Fig. 4). Parents in Ghana usually assume that their daughters will remain chaste until they are married and do not see the reasons to communicate with them about sexuality issues. Parents in Africa will rather trust teachers to talk about issues of abstinence and contraceptive use with their children in school [63; 64]. In Ghana, sexuality issues are not also discussed in church because the church is regarded as a holy place [63]. Studies in some African countries that explored mother–daughter communication about sexual maturation, abstinence and unplanned pregnancy revealed that mothers felt that it was a taboo to communicate with daughters about sexuality issues [63; 64]. Parents will always feel disappointed when they assume that their children will automatically avoid premarital sex even if they are not educated and warned of the dangers associated with it. Many parents lacked the requisite knowledge on such communications and were therefore uncertain on what to tell their children [64]. In the USA, some federal governments have imposed Abstinence-Until-Marriage Education programmes on their citizens [65]. However, data have consistently shown that half of high school students have had sex, which is why all young people need the information, skills, and access to services that will help them make and carry out informed, responsible decisions [65]. Parents have the responsibility to teach their adolescent children how to deal with sexual problems confronting them by educating them on what they need to do to avoid risky sexual behaviours [63]. It is better for parents to take a keen interest in the sexual maturations of their children rather than feel disappointed when they become pregnant outside of wedlock. Whether mothers have knowledge of abortion policies in Ghana and are ready to send their daughters to health facilities for safe abortion services remains to be explored by researchers. Evidence available shows that sex education continues to generate debates and controversies around the world [65]. Notwithstanding, many researchers have found more advantages for educating the girl child on sexuality issues [66, 67]. Parents must be educated about sex education and know what to tell their children during sex education. Mothers must also be educated on safe abortion services in Ghana so that they can help their children by educating and seeking the appropriate safe abortion service for them in health facilities or acquiring the right medications for them.

### A desire to pursue education

**Fig. 5:**
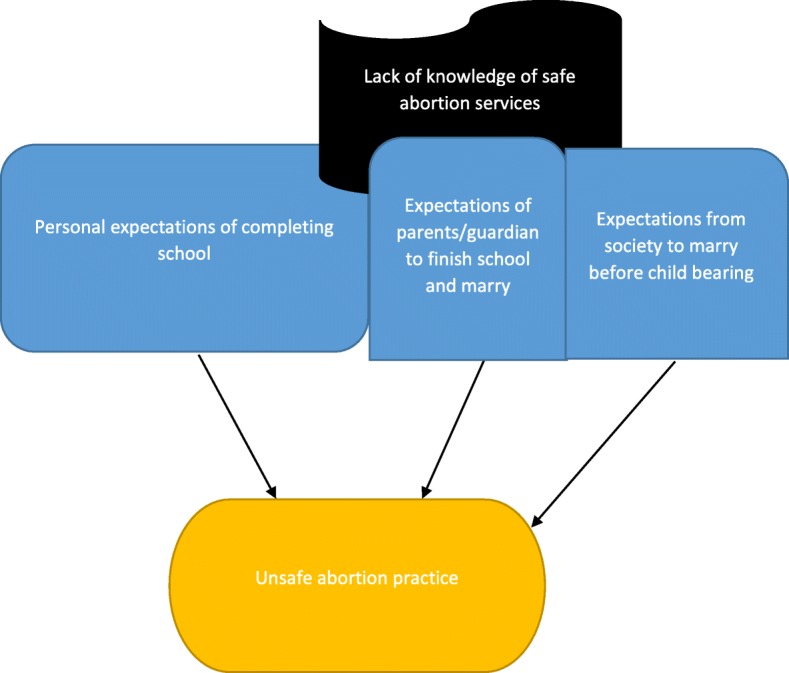
Desire to pursue education as cause of unsafe abortion practices. Source: conceptualised by authors (2018). This figure represents the seventh thematic result of this study with heading ‘Desire to pursue education’

A desire to pursue education was a contributing factor to unsafe abortion practices by young women in senior high and tertiary institutions in Ghana (Fig. 5). In Ghana, a girl child who gets pregnant in junior high and senior high school is likely to drop out of school. There are no marked differences in the proportion of males and females attending school up to age 16 [62]. However, there are substantially higher proportions of males than females attending school beyond age 16 [62]. Pregnancy and poverty have been mentioned by some researchers who studied dropout rate in Northern Ghana as contributing factors to drop out rate in the region [68]. Majority of patient participants (94%) were unemployed (Table 2). These patient participants probably hoped to find employments after school and therefore did not want an unwanted pregnancy to shatter their dreams. Though the influence of education desire on unsafe abortions has not been studied, one can assume that pupils and students at various levels of education in Ghana preferred to abort their pregnancies even if it will mean resorting to unsafe means, in order to continue being in school. This usually happens because the future of these students usually looked bleak without education. There are many calls by civil society groups for educational institutions to allow women who get pregnant to continue being in school if their conditions will allow. These human right considerations will have to be considered by every institution in Ghana to prevent drop out from school as a result of pregnancy. Education on contraception should also be improved in schools to prevent unwanted pregnancies among pupils and students.

### Recommendations for improved safe abortion practice in Ghana

The following recommendations are suggested based on findings in this research.

1. GHS and Ministry of Health (MOH) should adopt evidence-based public health approaches in educating women and the general public on the safe abortion policy of Ghana. This approach should concentrate on reducing maternal mortality and morbidity and harm reduction as pioneered in Uruguay.
2. MOH and Ghana Health Service (GHS) should disseminate information on its 2003 abortion policy amendment to health professionals and health programme managers.
3. Young people should be educated about sex and relationships by parents and schools.
4. The regimen for medical abortion should be publicised to replace the ineffective and dangerous unsafe abortion methods practiced by young women.
5. Concepts such as telemedicine need to be explored by health authorities in Ghana to improve implementation of Ghana’s safe abortion policy.
6. Task shifting to personnel such as nurses, midwives and pharmacist other than doctors only can help reduce maternal mortality and morbidity that occur because of unsafe abortions in Ghana.
7. Safe abortion information telephone hotlines such as Aunty Jane in Malawi and Kenya and Ms. Rosy in Nigeria can be utilised in Ghana in providing quick and adequate information on abortion to young women who may be considering that option.

## Conclusion

Several factors are responsible for unsafe abortion practices in Ghana. In this study, women cited lack of knowledge on the abortion law and safe abortion services, socio-economic conditions, cultural and religious beliefs, stigma of unplanned pregnancy, a desire to bear children only after marriage, avoidance of parental disappointment and resentment, and a desire to pursue education as reasons for practising unsafe abortions. Education on the Ghanaian safe abortion policy is a necessary step if the goal of avoiding preventable death from unsafe abortions is to be achieved. Curricula changes, continuous professional developments and public health education strategies are necessary to enlighten both health educators, students and the general public on the safe abortion policy of Ghana. A good knowledge by health teachers could eventually trickle down to students and the general public. Other teachers in senior high schools, colleges and universities should also be involved in the education on safe abortion services in Ghana so that they can in turn educate their students. Though further amendments and clarifications within the law are desirable, this may not happen any time soon. A liberal interpretation of the current law should be encouraged in Ghana. Measures such as Aunty Jane, Ms. Rose and Women Help Women programmes can be publicised to reduce maternal morbidity and mortality that results from unsafe abortions in Ghana. The regimen for medical abortion needs to be publicised to replace the ineffective and dangerous concoctions that were resorted to by women. Results of this study should be interpreted with care to populations not represented in this study. Further qualitative and quantitative studies covering all other regions in Ghana are recommended to explore the topic further. The views and perceptions of parents and other community groups could also be studied in a bid to provide a more complete view of contributing factors to unsafe abortion practices in Ghana.

## Additional file

Additional file 1: Guide for key informant interviews and fgd**.** This is the interview guide that was used for key informant interviews and focus group discussions. (DOCX 22 kb)
