# Supplementary material for: Contributing factors to unsafe abortion practices among women of reproductive age at selected district hospitals in the Ashanti region of Ghana
Source: BMC Womens Health. 2019 May 3;19:60. doi: 10.1186/s12905-019-0759-5 (PMC6500035; doi:10.1186/s12905-019-0759-5)
Supplement: Supplementary file 1 — Guide for key informant interviews and fgd. This is the interview guide that was used for key informant interviews and focus group discussions. (DOCX 22 kb) [file 12905_2019_759_MOESM1_ESM.docx]

**Additional file 1**

**GUIDE FOR KEY INFORMANT INTERVIEWS AND FGD.**

This study is being conducted to find out factors that contributes to the practices of unsafe abortion among females at their reproductive age. This interview guide contains open ended questions. You are expected to kindly provide genuine answers to the questions. The information you provide is confidential and will be used only for the purposes of this study. If you have any question, do not hesitate to ask the researchers. Your cooperation and participation until the completion of the interview or FGD is very necessary for the successful completion of the study. However your participation in this study is entirely up to you. You will not be penalized in anyway by refusing to participate.

**SECTION A: socio-demographic data**

**Kindly tick as appropriate:**

1. Age of respondent.

a. 15 – 19years

b. 20 – 24years

c. 25 – 29years

d. 30– 34years

e. 35– 39years

f. 40-44 years

g. 45-49 years

h. 50-54 years

i. 55-59 years

j. 60-64 years

k. 65-69 years

l. 70-74 years

g. Other…………..

1. Educational status
2. No formal Education
3. Nursery/Kindergarten
4. Primary school
5. Junior high school/JHS
6. Senior high school/SSS
7. Tertiary
8. Occupation
9. Employed
10. nurse,
11. doctor,
12. police
13. Trader
14. Pastor
15. Seamstress/tailor
16. Driver
17. Farmer
18. Hair dresser
19. Food vender
20. Unemployed
21. House wife
22. Student
23. Others………………
24. Religion
25. Islam
26. Christianity
27. Traditional Religion
28. Any other religion……………..

5. Marital Status

a. Single

b. Co-habitation

c. Married

d. Divorced

e. Widowed

f. Separated

g. Any other……………

6. Number of Children

a. None

b. One

c. Two

d. Three

e. Four

f. other, state…………..

**INTERVIEW SECTION**

**SECTION B:** **Knowledge on safe abortion practices**

7. Did you try aborting your pregnancy through an unsafe means? Yes……No………

8. Do you know what abortion is?

8. Describe what you know about abortion.

10. Explain what you know about the abortion law in Ghana.

11. What do you know about the abortion policy in Ghana?

Describe.

**SECTION C: Knowledge on unsafe abortion**

12. What do you know about unsafe abortions?

Describe.

13. Are you aware of the complications of unsafe abortions?

Describe the complications you know.

14. Which unsafe abortion methods did you use in attempting to abort your pregnancy. Describe.

**SECTION D: factors that influence unsafe abortion practices**

15**.** Have you ever performed abortion before this current one?

16. Why did you try aborting this pregnancy?

17. Did any financial difficulty influence your decision to terminate this pregnancy?

If yes, describe.

18. Describe your knowledge of safe abortion services in your community or country.

19. Describe situations or circumstances that made you practice unsafe abortion.

20. Describe reasons that prevented you from keeping your pregnancy.

21. Describe any impact of your pregnancy for which you decided to have an unsafe abortion.

22. Describe any impact/influence of relationships with friends and family or society on your decision to have an unsafe abortion.

23. Describe any other social or economic issues that influenced your decision to have an unsafe abortion.

**SECTION E: religious factors that influence unsafe abortion practices**

18. How does your religion perceive abortion?

Describe

19. How does your religion perceive the use of contraceptives?

Describe

20. Does your religion permit the practice of abortion?

If yes, explain your reason

If no, explain your reason

21. Describe your religious beliefs about abortion.

**SECTION F:** **cultural factors that influence unsafe abortion practices**

21. How does your community perceive the practice of abortion?

22. How does your society perceive pregnancy outside wedlock (from single women)?

Describe

23. Does your culture permit the practice of abortion?

If yes, explain why.

If no, explain why.

24. Describe your cultural beliefs about abortion.

**SECTION G: Additional questions for midwives, medical officers and religious leaders:**

Interviewee is (a) Midwife, (b) Medical Officer, (c) Religious leader

24. Why do you think young women attempt to abort their own pregnancies?

25. Do you think the culture and religion here accepts or rejects safe abortions. Describe your reason.

26. Does the community here accept safe abortion practices? Describe the reason for your answer.

27. Does the community here accept abortion in any form? Describe the reason for your answer.

28. Will you readily support any woman who wants to have a safe abortion in your facility?

If yes describe reasons

If no describe reasons

29. Are you aware of Ghana’s abortion law and policy? Describe.

30. Does you religion or culture support safe abortion services for women?

31. Do you have any other issues about abortion that you feel like sharing? Kindly describe.
